# Genetic Tracing of *Jatropha curcas* L. from Its Mesoamerican Origin to the World

**DOI:** 10.3389/fpls.2017.01539

**Published:** 2017-09-07

**Authors:** Haiyan Li, Suguru Tsuchimoto, Kyuya Harada, Masanori Yamasaki, Hiroe Sakai, Naoki Wada, Atefeh Alipour, Tomohiro Sasai, Atsushi Tsunekawa, Hisashi Tsujimoto, Takayuki Ando, Hisashi Tomemori, Shusei Sato, Hideki Hirakawa, Victor P. Quintero, Alfredo Zamarripa, Primitivo Santos, Adel Hegazy, Abdalla M. Ali, Kiichi Fukui

**Affiliations:** ^1^Department of Biotechnology, Graduate School of Engineering, Osaka University Osaka, Japan; ^2^Plant Bioengineering for Bioenergy Laboratory, Graduate School of Engineering, Osaka University Osaka, Japan; ^3^Kobe Food Resources Education and Research Center, Graduate School of Agricultural Science, Kobe University Hyogo, Japan; ^4^Arid Land Research Center, Tottori University Tottori, Japan; ^5^The Center for International Affairs, Tottori University Tottori, Japan; ^6^Graduate School of Life Sciences, Tohoku University Miyagi, Japan; ^7^Kazusa DNA Research Institute Chiba, Japan; ^8^INIFAP-Campo Experimental Bajío Guanajuato, Mexico; ^9^INIFAP-Campo Experimental Rosario Izapa Chiapas, Mexico; ^10^College of Agriculture, University of the Philippines Los Banos Laguna, Philippines; ^11^Genetic Engineering and Biotechnology Research Institute, University of Sadat City Sadat City, Egypt; ^12^Faculty of Agriculture, Shambat, University of Khartoum Khartoum, Sudan; ^13^Graduate School of Pharmaceutical Science, Osaka University Osaka, Japan

**Keywords:** *Jatropha*, biofuel plant, genetic diversity, center of origin, genetic resources

## Abstract

*Jatropha curcas* L. (Jatropha), a shrub species of the family Euphorbiaceae, has been recognized as a promising biofuel plant for reducing greenhouse gas emissions. However, recent attempts at commercial cultivation in Africa and Asia have failed because of low productivity. It is important to elucidate genetic diversity and relationship in worldwide Jatropha genetic resources for breeding of better commercial cultivars. Here, genetic diversity was analyzed by using 246 accessions from Mesoamerica, Africa and Asia, based on 59 simple sequence repeat markers and eight retrotransposon-based insertion polymorphism markers. We found that central Chiapas of Mexico possesses the most diverse genetic resources, and the Chiapas Central Depression could be the center of origin. We identified three genetic groups in Mesoamerica, whose distribution revealed a distinct geographic cline. One of them consists mainly of accessions from central Chiapas. This suggests that it represents the original genetic group. We found two Veracruz accessions in another group, whose ancestors might be shipped from Port of Veracruz to the Old World, to be the source of all African and Asian Jatropha. Our results suggest the human selection that caused low productivity in Africa and Asia, and also breeding strategies to improve African and Asian Jatropha. Cultivars improved in the productivity will contribute to expand mass commercial cultivation of Jatropha in Africa and Asia to increase biofuel production, and finally will support in the battle against the climate change.

## Introduction

*Jatropha curcas* L. (Jatropha) is a shrub that produces non-edible seed oil suitable for biodiesel fuel. The oil content ranges from 30 to 50% of its seed weight (Heller, 1996; Kumari et al., 2009). Jatropha has a potential to reduce the consumption of fossil fuel and carbon dioxide emissions (Bailis and Baka, 2010; Bahadur et al., 2013). Jatropha is drought tolerant, thereby its biofuel production could avoid competition with food crops (Fairless, 2007; Bahadur et al., 2013). Saving of carbon emissions depends on how biofuels are produced. Growing perennial plants on degraded and/or fallow fields for biofuel production has an advantage of sustained greenhouse gas reduction compared with converting forests or grasslands to produce annual crop-based biofuels (Fargione et al., 2008; Searchinger et al., 2008).

Large Jatropha plantations have been planned worldwide, particularly in the semiarid and arid areas of African and Asian countries by using seeds derived from local plants (Fairless, 2007; Von Maltitz and Setzkorn, 2013). Many commercial plantations, however, have not resulted in as high yields as were anticipated because of the limited productivity (Sanderson, 2009; Singh et al., 2014; Van Eijck et al., 2014; Von Maltitz et al., 2014). Breeding high-yielding Jatropha varieties is in its infancy so far (Divakara et al., 2010). Most of previous studies showed that genetic diversity of Jatropha is low in Africa, Asia and South America, but higher in Mesoamerica, including Mexico and Central America (Basha and Sujatha, 2007; Basha et al., 2009; Pamidimarri et al., 2010; Rosado et al., 2010; He et al., 2011; Montes et al., 2014; Montes Osorio et al., 2014; Siju et al., 2016). The low genetic diversity of African and Asian accessions has limited the potential for successful breeding by using local resources. The challenge now is to develop well-adapted, high-yielding varieties that are suitable for a wide range of climate conditions in African and Asian countries, since only wide plantation with high oil-production can guarantee a good supply for biofuel. Characterization and preservation of a diverse collection of Jatropha accessions, including those from Mesoamerica, is the key step to develop them.

Mesoamerica, especially Mexico, has been shown to possess high genetic variations of Jatropha and was assumed to be its place of origin (Makkar and Becker, 2009; Dias et al., 2012). Mexico has not only toxic Jatropha varieties, but also non-toxic ones, suggesting that Mexico might be also the domestication center of Jatropha (Dias et al., 2012). Recent studies described that Chiapas, the southernmost state of Mexico bordering Guatemala, might be the center of Jatropha biodiversity (Ovando Medina et al., 2011; Pecina Quintero et al., 2011, 2014; Salvador Figueroa et al., 2015). However, a comprehensive study is required to obtain more evidences for this conclusion.

It has been widely approved that Jatropha was brought by Portuguese from Mesoamerica to Cape Verde Islands, and then brought to Africa and Asia (Heller, 1996), while the transmission route in Mesoamerica is unknown and the genetic background of African and Asian Jatropha in the world population remains unclear. To improve the traits in the breeding goal of Jatropha in Asia and Africa, it is of great significance not only to identify diverse genetic resources but also to unveil the ancestral genotype of African and Asian Jatropha when shipped from Mesoamerica.

In this study, 59 simple sequence repeat (SSR) markers and eight retrotransposon-based insertion polymorphism (RBIP) markers were applied to assess the genetic diversity of 246 Jatropha accessions from eight Mexican states (Chiapas, Guerrero, Michoacan, Morelos, Oaxaca, Tabasco, Veracruz, and Yucatan), Guatemala and countries of Africa and Asia. We also evaluated the genetic variability of accessions from each of nine regions in Chiapas, to identify the center of origin. We further identified the voyage from Chiapas to the Old World by tracing the progenies in Mesoamerica sharing the same ancestors with nowadays African and Asian accessions. Human selection causing a low genetic basis in Africa and Asia is discussed. Finally, we propose strategies to improve African and Asian Jatropha, in order to increase the phenotypic performance including productivity, which will lead us to relieve from the climate change by mass production of the biofuel.

## Materials and Methods

### Plant Materials

A worldwide collection of 246 Jatropha accessions in this study consisted of 207 accessions from Mesoamerica (198 from Mexico and nine from Guatemala), seven from Africa, and 32 from Asia (Supplementary Table 1; see **Figure 3** for the locations of Mexican states and regions in the state of Chiapas). The Mexican accessions and seven Guatemalan accessions were obtained from the collection of the Instituto Nacional de Investigaciones Forestales, Agropecuarias (INIFAP, Mexico). Seven Philippine accessions were obtained from the collection of UPLB originating in four provinces of Luzon and Mindanao islands. Twenty-one Vietnamese accessions were obtained from plants grown in Quang Tri Province. Sudanese and Egyptian accessions were obtained from the University of Khartoum and University of Sadat City, respectively. Other accessions were the same as those used in our previous study (Sato et al., 2011). Jatropha sampling and transferring to Japan were performed in compliance with the Nagoya agreement.

### DNA Markers

Over 500 SSR markers were developed by designing primer pairs surrounding SSR sequences identified by the genomic database of Asian accessions based on our whole genome sequencing project (Sato et al., 2011; Hirakawa et al., 2012). Effective markers were selected from them based on clear polymorphisms and low number of null alleles. Eight RBIP markers employed in this study were developed from members of the copia-type families identified in the genomic database (Alipour et al., 2013). All of them were expected to have retrotransposed more recently than other members. Primers were designed for both flanking (FLK) sequences and long terminal repeats (LTRs). Retrotransposon insertion at each locus was shown by combining primers designed from the FLK sequences at both sides and primers designed from LTR sequences. Linkages between the markers were tested by TASSEL2.1 (Bradbury et al., 2007).

### DNA Extraction

Genomic DNA was extracted from Jatropha leaves with a DNeasy Plant Mini Kit (Qiagen, United States) according to the manufacturer’s instructions. The DNA samples were diluted to a final concentration of 0.35 ng/μl and stored at -20°C until use.

### Genotyping and Scoring

Polymerase chain reaction (PCR) for SSR markers was performed in a 10 μl total volume solution containing 1.4 ng of DNA template, 0.16 μl of SSR primer mix (25 μM each), 1× PCR buffer, 0.03 μM MgCl_2_, 0.8 μl dNTPs (2.5 mM), 0.08 U Bio-taq (5 units/μL), and Milli Q water. Touch-down amplification was performed as follows: 3 min hold at 94°C, followed by three cycles at 94°C for 30 s, and 68°C for 30 s, with the annealing temperature reduced by 2°C until 64°C every three cycles. Continuing with three cycles at 94°C for 30 s, 62°C for 30 s, and 72°C for 30 s, the annealing temperature was reduced by 2°C until 58°C every three cycles. A further 30 cycles of amplification was performed at 94°C for 30 s, 55°C for 30 s, and 72°C for 30 s. The PCR products were stored at -20°C until separation by polyacrylamide gel electrophoresis (PAGE). Amplified bands were stained with ethidium bromide. For markers that showed ambiguous bands, experiments were repeated until clear bands were observed by increasing annealing temperatures.

For RBIP markers, PCR was performed in a 5 μl total volume solution containing 0.7 ng of DNA template, 0.08 μl of primer mix (50 μM each), 1× PCR buffer, 0.03 μM MgCl_2_, 0.4 μl dNTPs (2.5 mM), and 0.08 U Bio-taq (5 units/μL), made to the volume with Milli Q water. PCR reaction was started with denaturation at 94°C for 2 min, followed by 35 cycles of 45 s at 94°C, 45 s at 55°C and 2 min at 72°C, continued with a final elongation step at 72°C for10 min. Amplified bands were stained with ethidium bromide. The PCR products were stored and analyzed like the SSR markers.

For all the SSR markers and RBIP markers, bands were individually scored (presence 1, absence 0). All data analyses were performed based on the genotype data matrix.

### Genetic Heterozygosity

Expected heterozygosity (*H*_E_) and observed heterozygosity (*H*_O_) show the portion of heterozygotes in populations. They are measures of the extent of genetic variation in a population. *H*_E_ is heterozygosity under Hardy–Weinberg equilibrium (HWE) by random mating of individuals (Nei, 1987). *H*_O_ is heterozygosity at the observed level. Differences between *H*_E_ and *H*_O_ are caused by inbreeding often as results of selection or the small population size. Inbreeding coefficient (*F*_IS_) is the value to estimate the level of inbreeding of the population. *F*_IS_ was calculated from *H*_E_ and *H*_O_ by the equation: *F*_IS_ = 1 -*H*_O_/*H*_E_. The larger *F*_IS_ shows the larger extent of inbreeding in a population. *H*_E_ and *H*_O_ were calculated with GenALEx 6.5 (Peakall and Smouse, 2006). Comparisons of the significances of *H*_E_ and *H*_O_ among populations were performed using Kruskal–Wallis one-way analysis of variance. Calculation of the *F*_IS_ and *P*-values, which show the significance of inbreeding, was performed with FSTAT version 2.9.3.2 (Goudet, 2001).

### Population Structure

Model-based clustering analysis was performed using Mesoamerican accessions or all accessions including African and Asian accessions with STRUCTURE 2.3.4 (Pritchard et al., 2000). The analysis was performed using the Markov chain Monte Carlo method based on 1 × 10^5^ iterations followed by a burn-in period of 5 × 10^4^ iterations. To determine the optimal number of populations (K), ΔK method was applied. K varied from 1 to 10, after 10 independent runs for each K, ΔK was calculated based on the rate of change of the log likelihood for K values between successive K values following Evanno et al. (2005). ΔK should have a clear peak at true value of K.

Principal coordinates analysis (PCoA) of all individuals was performed with GenALEx 6.5. The final results were shown as a three-dimensional plot (X, Y, and Z represent the three principal coordinates) of all the accessions.

### Phylogenetic Relationship

A neighbor-joining (NJ) tree of African and Asian accessions and non-admixed Mesoamerican accessions with more than 80% of the group membership in Mesoamerican structure analysis was constructed on the basis of genetic distances (Nei et al., 1983) with POPULATION 1.2.32 (Langella, 2002). The tree was drawn with Treeview 1.6.6. (Page, 1996) and Figtree 1.4.2. (Rambaut and Drummond, 2014). Genetic distances (Nei’s D_A_ distance) between groups (admixed accessions with less than 80% of the group membership in Mesoamerican structure analysis were excluded in the analysis) were also calculated with POPULATION 1.2.32.

### Number of Retrotransposons

Presence of each of the eight retrotransposons was judged by presence of the amplified band using a FLK primer and a LTR primer, and in the case of full-length members, also by absence of the band using FLK primers at both sides of the retrotransposon. According to the results, number of retrotransposons present in each accession was counted.

### Approximate Bayesian Computation (ABC) Analysis

To characterize the Mesoamerican ancestral genetic group of African and Asian accessions, we assumed that African and Asian Jatropha originated from different Mesoamerican genetic groups and then constructed scenarios. To decide which scenario is the best supported by data, scenarios were compared in DIYABC v2.1.0 (Cornuet et al., 2008, 2010).

### Phenotypic Variation

Number of inflorescences per plant, number of female flowers per plant, ratio of female to male flowers and seed yield (g) per plant of 100 representative accessions from seven Mexican states [Chiapas (57), Guerrero (4), Michoacan (4), Morelos (3), Oaxaca (7), Veracruz (20), and Yucatan (5)] were measured individually at the Rosario Izapa Experimental Farm, INIFAP (Chiapas, Mexico). Chiapas accessions were further sub-classified into those from six regions [Centro (14), Frailesca (8), Fronteriza (3), Sierra (4), Selva (2), and Soconusco (26)]. To compare the variation among accessions from central Chiapas (Centro, Fronteriza, Frailesca, and Sierra), peripheral areas of Chiapas (Selva and Soconusco), and other Mexican states, the mean value and standard deviation (SD) of each area were calculated.

## Results

### Genetic Diversity in Mesoamerica

To survey the worldwide genetic variation of Jatropha, 59 effective SSR markers that showed the intraspecific polymorphism were selected from more than 500 Jatropha SSRs. The mean percentage of the missing data of SSR markers was 0.79%. We also identified six full-length members with two LTRs showing 100% sequence identity with each other, as well as two solo LTRs displaying 100% sequence identity with the consensus sequence, from Jatropha copia-type retrotransposon families (Alipour et al., 2013) to use as RBIP markers. All of the eight RBIP markers showed the intraspecific polymorphism. The mean percentage of the missing data of RBIP markers was 1.37%. Primers for the SSR markers and RBIP markers used in our study were listed in Supplementary Tables 2, 3. There were no or low linkage between all the markers (data not shown).

The SSR markers yielded 221 polymorphic bands ranging from two to seven with an average of 3.75 alleles per marker in all accessions. Among them, 161 alleles were specific to Mesoamerica, whereas no allele was specific to Africa or Asia (Supplementary Table 1). The state of Chiapas had 47 specific alleles, which was the highest number among Mexican states and Guatemala. In Chiapas, 30 alleles were specific to the central areas (Centro, Altos, Fronteriza, Frailesca, and Sierra), while only one allele was specific to the peripheral areas (Norte, Selva, Soconusco, and Istomo-Costa) (Supplementary Table 1).

**FIGURE 1:**
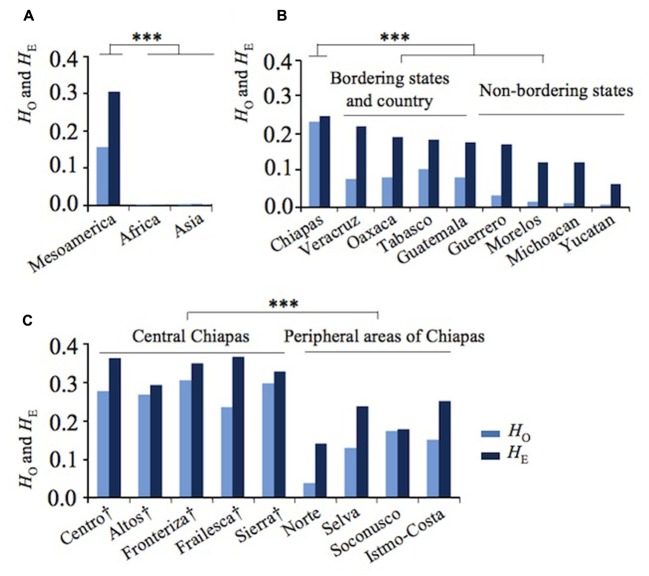
Genetic divergence of Jatropha in Mesoamerica. **(A)** Observed (*H*_O_) and expected (*H*_E_) heterozygosity in Mesoamerica, Africa, and Asia. **(B)** *H*_O_ and *H*_E_ in Mexican states and Guatemala. **(C)** *H*_O_ and *H*_E_ of regions in Chiapas states. Regions in central Chiapas covering the Chiapas Central Depression are shown by †. ^∗∗∗^*P* < 0.001.

Remarkably, 39 Asian and African accessions were almost monomorphic and homozygote in both kinds of markers, except for five Vietnamese accessions, each of which was heterozygote in a single marker. Negligible expected heterozygosity (*H*_E_) or genetic variation was observed in African and Asian accessions (*H*_E_ = 0.000 and *H*_E_ = 0.002, respectively) (**Figure 1A** and Supplementary Table 1). On the contrary, a significantly higher *H*_E_ value was obtained for Mesoamerican accessions (**Figure 1A** and Supplementary Table 1).

In Mesoamerica, the highest genetic variation was found in Chiapas, with a clear decreasing cline of *H*_E_ from Chiapas and its bordering states to non-bordering states (**Figure 1B**). These results showed that the highest genetic diversity of Jatropha exists in Chiapas.

To further narrow down the center of genetic variation, *H*_E_ was examined among the regions within Chiapas. Five regions in central Chiapas had significantly higher *H*_E_ values than four regions in the peripheral areas (*P* < 0.001, **Figure 1C**). Slight differences between expected and observed heterozygosity (*H*_E_ versus *H*_O_) were observed among the accessions from central Chiapas, whereas large differences between these statistics were detected among the accessions from the peripheral areas of Chiapas, other Mexican states, and Guatemala (**Figures 1B,C**). These resulted in lower inbreeding coefficients (*F*_IS_) in central Chiapas than in most of the peripheral areas of Chiapas, other Mexican states, and Guatemala (Supplementary Table 1). This may be due to human selection and/or decrease of effective population size in the areas other than central Chiapas. Interestingly, in Soconusco, the southernmost peripheral region of Chiapas, *F*_IS_ was the lowest, and in Sierra, one of the central regions, locating near to Soconusco, *F*_IS_ was the second lowest. It is likely that effect of the human selection was little in these regions, but further research would be required to prove it.

### Phenotypic Diversity in Mesoamerica

The phenotypic variation of four yield-related traits was evaluated for 100 Mexican accessions. The mean values and SDs of the accessions from central Chiapas were higher than those from the peripheral areas of Chiapas and other Mexican states (Supplementary Table 4). These suggest that Jatropha population in central Chiapas has the highest phenotypic variation in Mexico.

### Genetic Groups in Mesoamerica

**FIGURE 2:**
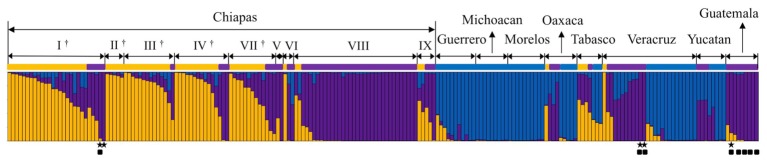
Model-based clustering (*K* = 3) of Mesoamerican accessions. Three genetic populations, Groups A, B and C, are indicated in orange, purple, and blue, respectively. Mesoamerican group of each accession is indicated with different group color. The origin of each accession is shown above (see Supplementary Table 1 for origins). I ∼ IX show regions in the state of Chiapas (I: Centro, II: Altos, III: Fronteriza, IV: Frailesca, V: Norte, IV: Selva, VII: Sierra, VIII: Soconusco, and IX: Istmo-Costa), and regions in central Chiapas covering the Chiapas Central Depression are shown by †. 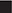 Eight accessions that classified into the African and Asian group (Supplementary Figure 2). 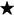 Accessions carrying all the eight retrotransposons.

**FIGURE 3:**
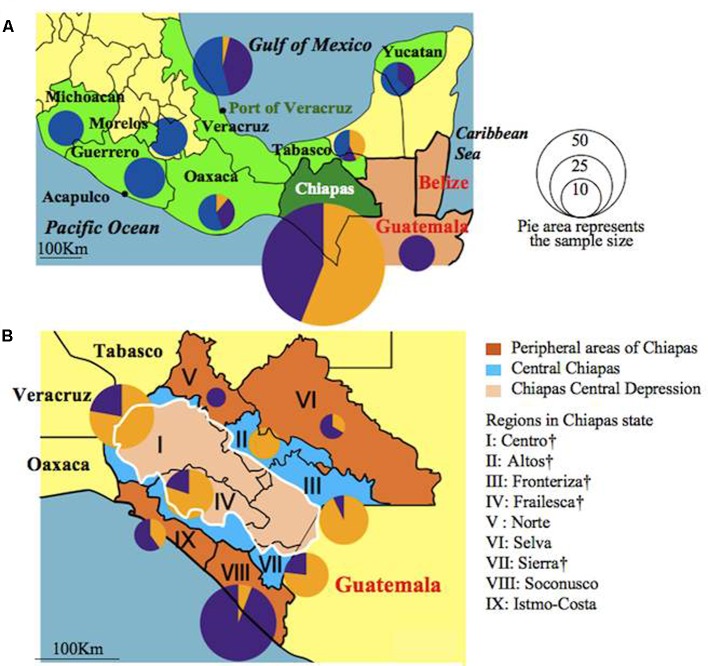
Distribution of accessions assigned to Groups A, B, and C in Mexican states, Guatemala **(A)** and Chiapas regions **(B)** illustrated as pie graphs. Sizes of the circles correspond to the sample sizes. Mexican states were colored green. Central Chiapas is colored blue and peripheral areas of Chiapas is colored brown. The Chiapas Central Depression is colored pink. Regions in central Chiapas covering the Chiapas Central Depression are shown by †.

The genetic constitutions of all the Mesoamerican accessions were examined by structure analysis. The ΔK value with respect to K (number of groups in the population) showed a peak when K equaled to 3 (Supplementary Figure 1A; see Population Structure). This indicates that the optical group number of all Mesoamerican accessions is 3, and then accessions from each region were classified into three clear genetic groups: A, B and C, by the model-based clustering analysis (**Figure 2**). The numbers and ratios of accessions assigned to each group in each Chiapas region, other Mexican states and Guatemala are presented in **Figure 3**. The distribution of the groups revealed a distinct geographic cline. Accessions from central Chiapas were mostly in Group A (orange). Accessions in Group B (purple) were mainly distributed in the peripheral areas of Chiapas and in neighboring states and countries, whereas accessions in Group C (blue) were mainly distributed in states distant from Chiapas (Guerrero, Michoacan, and Morelos). This geographical distribution of Jatropha genetic groups in Mesoamerica has not been reported before. The distribution of Group B is especially interesting, because it seems to surround the area of Group A.

The highest genetic variation and the lowest *F*_IS_ among the three groups were observed in Group A (Supplementary Table 5), which was as expected from the results of the previous section. Mean values and SDs of phenotypic variations in Groups A, B, and C were also calculated and compared. Accessions from Group A had higher mean values and variations of the four traits than those from Groups B and C (Supplementary Table 4).

### Mesoamerican Accessions Genetically Closest to African and Asian Jatropha

To estimate the ancestral genotypes of African and Asian Jatropha in their origin, we searched Mesoamerican accessions that are genetically close to them. We performed population structure and phylogenetic analyses on Jatropha including African and Asian accessions. The ΔK value with respect to K showed the maximum when K equaled to 3 (Supplementary Figure 1B), meaning that the optical group number of all the accessions is 3, and then they were classified into three genetic populations: Groups I, II, and III, by the model-based clustering analysis (Supplementary Figure 2). Group II mainly corresponds to Mesoamerican Group A, whereas Group III is composed of most of Mesoamerican accessions of Groups B and C, further indicating the distinct differences between Group A and other accessions in Mesoamerica. Group I comprises all the African and Asian accessions and eight Mesoamerican accessions: one from central Chiapas; two from Veracruz; and five from Guatemala. The genetic constitution of African and Asian accessions proved to be almost identical, indicating that they are the progenies of inbreeding through successive generations within limited ancestral genotypes. Interestingly, the eight Mesoamerican accessions in Group I were those classified into Group B in the previous section (**Figure 2**), and African and Asian accessions had the closest genetic and phylogenetic relationships with Group B (Supplementary Table 6). Similarly, accessions from the peripheral areas of Chiapas, Veracruz and Guatemala had the closest relationships with African and Asian accessions (Supplementary Table 7). Results of Approximate Bayesian Computation (ABC) analysis supported our conclusion that African and Asian accessions were derived from Group B accessions (Supplementary Figure 3).

**FIGURE 4:**
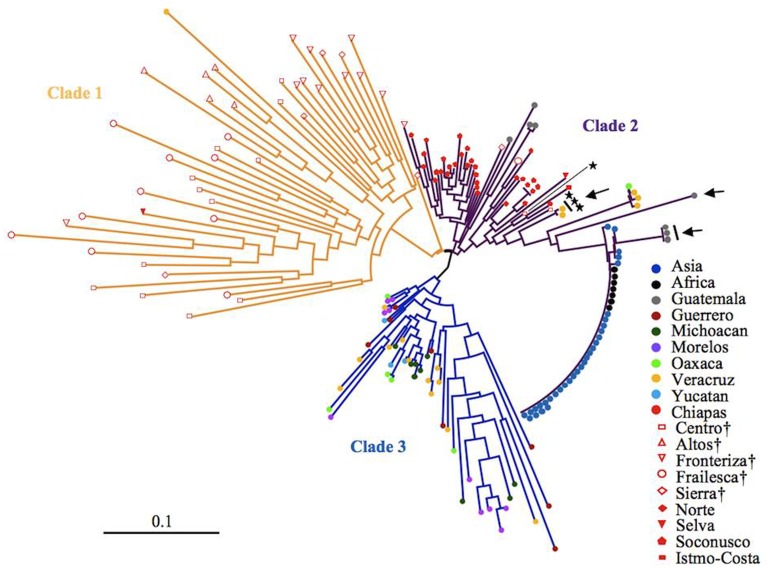
A neighbor-joining tree of non-admixed Mesoamerican accessions and African and Asian accessions. 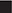 Seven accessions that genetically close to African and Asian group in Supplementary Figure 2. 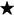 Accessions carrying all the eight retrotransposons. Regions in central Chiapas covering the Chiapas Central Depression are shown by †.

**FIGURE 5:**
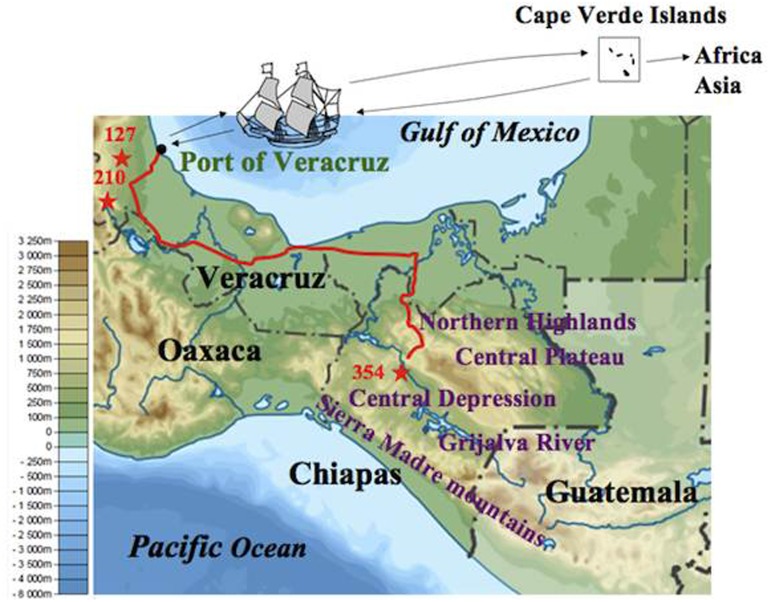
Jatropha’s voyage from the Chiapas Central Depression to the Old World. The map shows the altitudes of Chiapas and its surrounding areas. The background map is available from https://commons.wikimedia.org/wiki/File:Mexico_ topographic_map-blank.svg. The stars represent the sampling sites of three accessions that share the same ancestor of African and Asian Jatropha. The red line represents a current road between Veracruz and Chiapas.

After eliminating admixed accessions which showed < 80% of the membership of each Mesoamerican Groups (see **Figure 2**), a phylogenetic tree of African and Asian accessions and non-admixed Mesoamerican accessions was constructed. The tree showed three clades, and interestingly, all of Mesoamerican accessions in each of the three clades (clades 1, 2, and 3) belonged to each of the three Mesoamerican Groups (Groups A, B, and C in **Figure 2**), respectively. One (Guatemalan accession) of the eight Mesoamerican accessions that genetically close to African and Asian groups (see above and Supplementary Figure 2) was not included in the tree because of its admixed membership. Remaining seven Mesoamerican accessions were in the same clade with all African and Asian accessions in the phylogenetic tree (**Figure 4**). PCoA analysis also revealed a close relationship between these seven accessions and African and Asian ones (Supplementary Figure 4). These seven accessions are genetically closest to African and Asian Jatropha, and most likely share common ancestors with them. Because insertions of copia-type retrotransposons in the genome are irreversible, the presence/absence of them is a reliable indicator for determining parental lineages. Among the seven accessions, one from the Centro region (central Chiapas) and two from Veracruz shared all eight retrotransposons identified in the African and Asian accessions (**Figures 2**, **4**, Supplementary Figure 2 and Table 8). On the other hand, the other four accessions from Guatemala lacked one of the eight retrotransposons. This observation suggests that the genotype of the three accessions [No. 127 (Soledad de Doblado, Veracruz), 210 (Entrada a Independencia, Veracruz), and 354 (Suchiapa, Centro, Chiapas)] (**Figure 5**) is the closest to the ancestral genotypes of African and Asian Jatropha.

## Discussion

Aiming to identify the center of origin possessing the most diverse genetic resources of Jatropha that would contribute to breeding projects in Africa and Asia, we examined genetic diversity of a worldwide collection of Jatropha accessions. We found that Mesoamerican populations showed significantly higher genetic diversity than African and Asian populations, and the Chiapas population showed the highest. Low genetic variation of local accessions in Africa and Asia should restrict their phenotypic performance, which would have resulted in the limitation in breeding programs. These results are consistent with previous reports (Basha and Sujatha, 2007; Basha et al., 2009; Pamidimarri et al., 2010; Rosado et al., 2010; He et al., 2011; Montes et al., 2014; Montes Osorio et al., 2014; Ouattara et al., 2014; Yue et al., 2014; Guo et al., 2016; Montes and Melchinger, 2016; Siju et al., 2016). Exceptionally, Maghuly et al. (2015) showed the most diverse population in Cape Verde by using 907 accessions from three continents. It was probably because they used only 19 Mesoamerican (Mexican) accessions from limited collection sites. Note that they found that SNPs of some genes were mostly in Mexican accessions in the same paper.

We further narrowed down the center of diversity to central Chiapas. The main part of central Chiapas is consisted of the Chiapas Central Depression (Pons et al., 2016), which is an ecoregion defined by its vegetative and geographic features. The Depression, which extends over five regions in central Chiapas, is composed of hot and semiarid lowlands dominated by deciduous shrubs. It is sandwiched by the northern highlands, central plateau and the southern Sierra Madre Mountains. An east–west running Grijalva river divides the Depression. Besides, the northern and southern parts of Chiapas have the humid tropical climate where the rainfall is plenty. Averaged annual rainfall in some areas of these parts exceeds 3,000 mm, and there exists a large scale of the tropical rainforest. On the other hand, the central Chiapas has less humid climate, annual precipitation ranges from 750 to 1500 mm (Newton and Tejedor, 2011). The tropical rainforest in the peripheral areas of Chiapas may have prevented natural diffusion of Jatropha from the central part, because the optimal rainfall for Jatropha is from 1000 to 1500 mm, and too much rain and humidity will provoke fungus and high rainfall might cause root damage (Ouwens et al., 2007). Isolated geographical and climate situations of the Chiapas Central Depression may have favored the evolution of drought-adapted plant species. The overlap among the Jatropha centers of genetic and phenotypic variation, as well as the pronounced geographical and climatic conditions, suggests that the Chiapas Central Depression is most likely the center of origin of Jatropha.

Chiapas Central Depression has the most diverse genomic resources, thus unexplored useful natural variants or mutants can be expected there. It would be the most suitable region to search Jatropha resources for breeding, which will contribute to enriching the pool of Jatropha for further genomic study. By using DNA markers derived from the genomic sequencing of an Asian accession (Sato et al., 2011), we could anchor the most diverse and various materials of Jatropha. Further genomic sequencing of Mexican accessions will lead to identifying more number of genome-wide effective DNA markers, which will encourage genomic selection or genome wide association studies (GWASs) of Jatropha from its center of origin by combing with phenotypic data over years. Diverse genomic resources can also be used for the study on the interactions among genes and/or genomic regions. Our identification of the center of diversity not only pointed out the center of origin, but also will inspire further genomic study of Jatropha.

We found genetic grouping (Groups A, B, and C) that correlated with geographic location in Mesoamerica, which has not been reported previously. Our results suggest that Group A, which distributes in central Chiapas represents the original genetic group. Specific genotypes of Group A might have spread from the Chiapas Central Depression to bordering areas, and subsequently formed distinct genotypes that comprise Group B and then C, according to their geographical distribution. We showed that African and Asian accessions were derived from Group B, rather than Group A or C. Further ABC analysis would be useful to make this conclusion more convincing.

In our study, we found some Group B accessions from Veracruz and Guatemala were genetically close to African and Asian accessions (Supplementary Table 7). Because Veracruz and Guatemala locate in the opposite direction (west and east, respectively) of the Chiapas Central Depression, the Jatropha in Group B from the peripheral areas of Chiapas, Veracruz and Guatemala might have experienced some genetic diffusion and exchange before evolving into the ancestral genotype of African and Asian Jatropha. This exchange would have involved human selection of the specific Jatropha genotype with advantageous phenotypes across these regions.

Identification of the two Veracruz accessions that genetically close to African and Asian Jatropha showed that Veracruz was an important junction between Mesoamerica and the Old World for the Jatropha transmission. Veracruz has been a major port facing the Gulf of Mexico since 1519, and there was no comparable port on the Caribbean coast of Guatemala. Moreover, the routes from Veracruz to other places in Mexico were gradually developed as the importance of the port increased. During the 16th and 17th centuries, Veracruz was one of the three major American ports for transatlantic trade. There was a fixed route between the Cape Verde Islands and Veracruz for slave transport from Africa (Thomas, 2006; Bryant, 2014). It is thus reasonable to speculate that Jatropha was taken to the Cape Verde Islands and then to Africa via the Port of Veracruz.

Interestingly, the two Veracruz accessions that carry all the eight retrotransposons were collected at Soledad de Doblado and Entrada a Independencia of Veracruz, both of which are within 40 and 100 km from the Port of Veracruz, respectively. A Chiapas accession that is genetically close to the African and Asian group but also carries all the retrotransposons was collected at Suchiapa region in the Chiapas Central Depression (**Figure 5**). It is also noteworthy that this Chiapas accession is in Group B, even though Group A represents the major genetic background there (**Figure 3**). The two Veracruz accessions also belong to Group B. The absence of genetically similar accessions between central Chiapas and the port of Veracruz excludes a gradual natural expansion by seeds and favors the human-mediated spread of Jatropha from central Chiapas to the Port of Veracruz. These also suggest that there were selections when people brought Jatropha from central Chiapas. One important question is as follows: who first selected the ancestral African and Asian Jatropha in central Chiapas? The Spanish conquistadors normally did not enter the Chiapas Central Depression, where no mineral resources were expected to occur (Ochiai, 1991). In addition, there exist various non-Spanish local names of Jatropha in Chiapas and Veracruz (unpublished results by Ando). Therefore, it is probable that the indigenous people of Mexico selected and carried a limited genotype of Jatropha seeds or cuttings in central Chiapas and transported them to the area close to the Port of Veracruz, to be the ancestor of two Veracruz accessions. Then they would have been transported to Africa and Asia via the Cape Verde Islands by Portuguese from the Port of Veracruz (**Figure 5**) (Heller, 1996).

As a limited number of genotypes would have been brought to the Old World by Portuguese galleons, crosses could have occurred only among small populations in the Old World plantings, yielding very low genetic variation. This breeding bottleneck represents a typical example of the founder effect and is probably the major cause of the failure of Jatropha breeding in African and Asian countries. In addition, accumulation of mildly deleterious genes exhibited in the homozygous state by inbreeding might show inbreeding depression. In fact, one of the Veracruz accessions (No. 127), which has been suggested to share a common ancestor with African and Asian accessions as discussed above, had low values for the four yield-related traits (number of inflorescences per plant: 3; number of female flowers per plant: 18; ratio of female to male flowers: 0.06 and seed yield per plant: 15.10 g) comparing with those in each region and group in Mexico (Supplementary Table 4), suggesting that the low seed-yield of African and Asian accessions is genetically determined. Moreover, Mesoamerican people seemed not to have placed any value on the seed-yielding potential of Jatropha as they selected and brought Jatropha seeds or cuttings from Chiapas to Veracruz for use as live fences and medicines (Duke, 1985; Prasad et al., 2012; Agbogidi et al., 2013), and these traditional applications are still widespread among Mesoamerican inhabitants even nowadays. Fences and medicines are the likely reasons why the Portuguese shipped the plant to Africa and Asia (Sabandar et al., 2013). Since then, seed productivity did not have a high priority in most part of Africa and Asia until recently. After arrival to Africa and Asia, epigenetic mutations might have occurred to adapt to the local climate within 100s of years, which have caused minor phenotypic variations, as discussed in previous reports (Montes Osorio et al., 2014; Yue et al., 2014).

Based on our findings that the genetic basis of African and Asian accessions is Group B in Mesoamerica and that Group A shows the highest genetic diversity in Mesoamerica, we propose the following strategies for genetic improvement in African and Asian Jatropha. First, make wide crosses between elite lines from each country in Africa and Asia, which should have their epigenetic adaptations to the climatic conditions, and accessions of Group A from central Chiapas, which are genetically distant from African and Asian Jatropha and have the largest genetic diversity. Not only hybrid vigor, but also some extent of phenotypic variations which are derived from the highest heterozygosity of Group A, would be expected in F_1_ progenies. Second, select elite F_1_ or F_2_ progenies by phenotypic performance. Finally, use vegetative propagation (Behera et al., 2014) of selected progenies to preserve excellent lines in the heterozygous status. This strategy would be better than crossing among African and Asian Jatropha, or than just transplanting the Mexican one, because of the higher genetic diversity, heterozygosity and climate adaptability. Production of Jatropha cultivars with high productivity in Africa and Asia will help in the mass commercial cultivation of Jatropha there. High production of Jatropha seeds can ensure a stable and enough supply of biofuel, which will help in the battle against the climate change.

## Author Contributions

HL wrote the manuscript; HL, ST, KH, and KF discussed the results, organized and revised the manuscript; MY supported and conducted some statistical analyses; HS and NW commented the manuscript and supported the experiment; HL, AA, and TS performed the genotyping experiment; AT, HTs, TA, and HTo suggested and helped the data collection and analyses. SS and HH designed SSR markers and performed part of the marker analysis; VQ and AZ collected Mexican accessions and provided the phenotype data of Mexican accessions; PS collected and provided Philippines accessions; AH provided one Egyptian accession; AMA provided two Sudanese accessions. KF organized the project.

## Conflict of Interest Statement

The authors declare that the research was conducted in the absence of any commercial or financial relationships that could be construed as a potential conflict of interest. The reviewer NO declared a shared affiliation, with no collaboration, with one of the authors MY to the handling Editor.
