# Supplementary material for: Genetic Tracing of Jatropha curcas L. from Its Mesoamerican Origin to the World
Source: Front Plant Sci. 2017 Sep 7;8:1539. doi: 10.3389/fpls.2017.01539 (PMC5594977; doi:10.3389/fpls.2017.01539)
Supplement: Supplementary file 1 [file Data_Sheet_1.pdf]

Supplementary Material

Genetic tracing of *Jatropha curcas* L. from its Mesoamerican origin to the world

Haiyan Li[1], Suguru Tsuchimoto[2], Kyuya Harada[2], Masanori Yamasaki[3], Hiroe Sakai[2], Naoki Wada[2], Atefeh Alipour[1], Tomohiro Sasai[1], Atsushi Tsunekawa[4], Hisashi Tsujimoto[4], Takayuki Ando[5], Hisashi Tomemori[4], Shusei Sato[6], Hideki Hirakawa[7], Victor Pecina-Quintero[8], Alfredo Zamarripa[9], Primitivo Santos[10], Adel Hegazy[11], Abdalla Mohamed Ali[12], and Kiichi Fukui[13, *]

* Correspondence:
Kiichi Fukui
kfukui@bio.eng.osaka-u.ac.jp

This supplementary material contains:
4 supplementary figures
8 supplementary tables

1 Supplementary Figures and Tables

1.1 Supplementary Figures

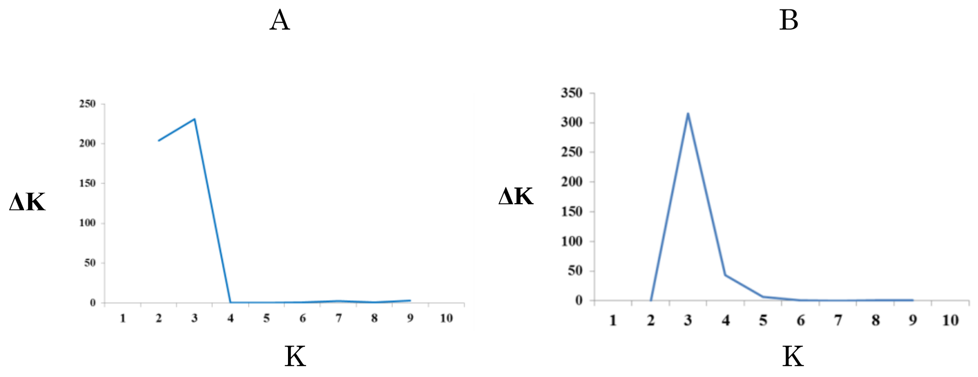

Supplementary Figure 1. Results of the Structure analysis to infer population numbers in Mesoamerican accessions (A) and all accessions (B) by using ΔK method, which are used for the analyses in Figure 2 and Supplementary Figure 2, respectively.

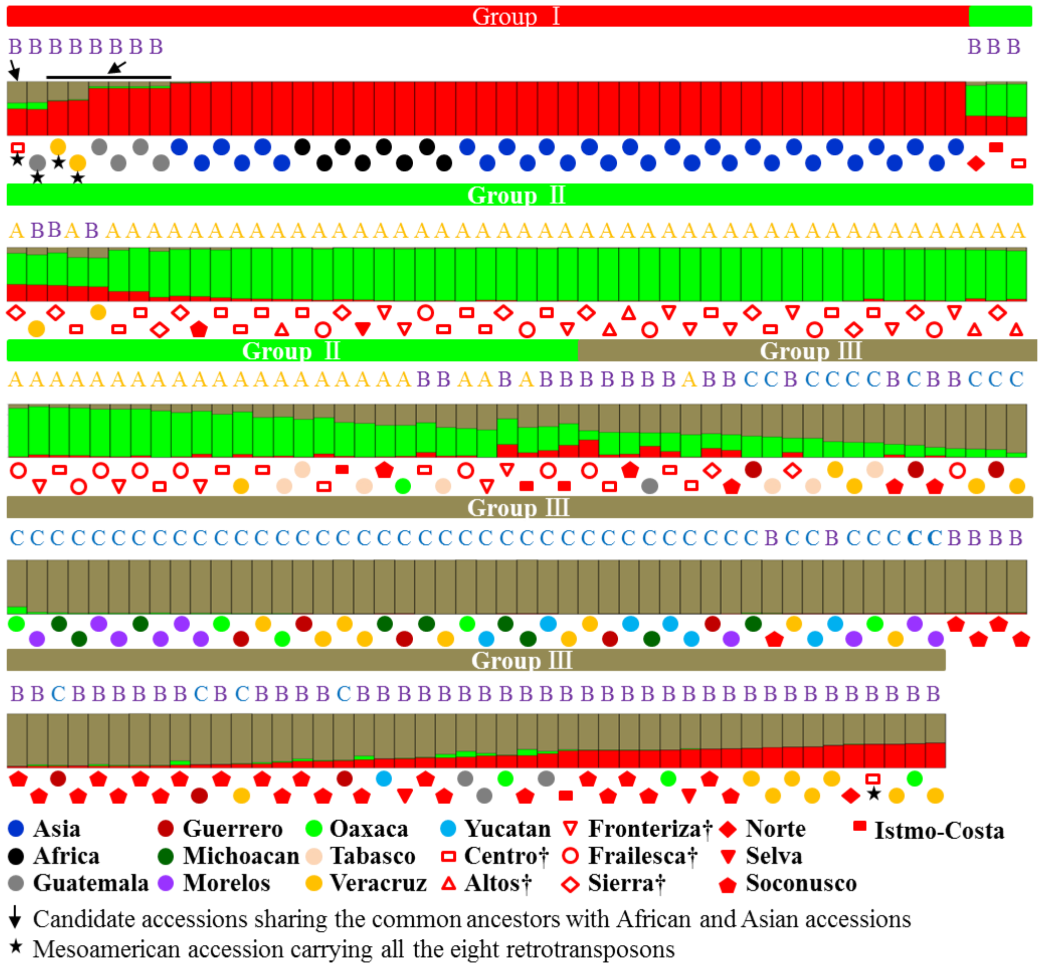

Supplementary Figure 2. Model-based clustering (K = 3) of Mesoamerican, African and Asian accessions. Three Groups I, II, and III are indicated in red, green and grey colors, respectively. Results of the grouping in Mesoamerican accessions (see Figure 2) are also shown as A, B, and C.

A

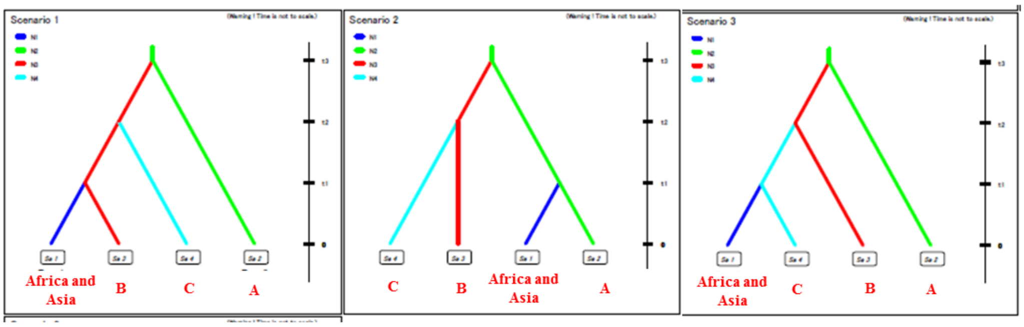

B DIYABC prior and posterior distributions for demographic parameters

	Prior distribution			Posterior distribution				
Parameter	Distribution	Minimum	Maximum	Mean	Median	Mode	q5%	q95%
N1	Uniform	10.0	10000.0	2.05E+02	1.16E+02	7.87E+01	3.95E+01	4.85E+02
N2	Uniform	10.0	10000.0	2.66E+03	2.57E+03	2.52E+03	1.38E+03	4.01E+03
N3	Uniform	10.0	10000.0	1.22E+03	9.88E+02	8.24E+02	3.75E+02	2.92E+03
N4	Uniform	10.0	10000.0	2.38E+03	2.04E+03	1.87E+03	7.87E+02	5.25E+03
t1	Uniform	10.0	10000.0	5.05E+02	4.27E+02	3.03E+02	9.56E+01	1.10E+03
t2	Uniform	10.0	10000.0	1.63E+02	1.43E+02	1.23E+02	6.40E+01	3.12E+02
t3	Uniform	10.0	10000.0	4.07E+02	2.63E+02	2.36E+02	9.96E+01	1.14E+03

C

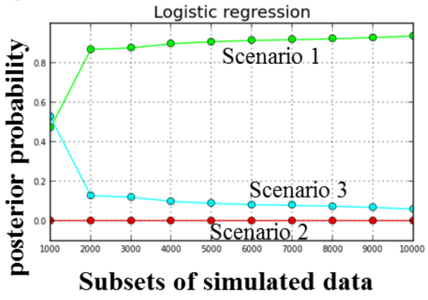

D Type I and Type II error rates for each scenario

	Type II error			Type I error
	Scenario1	Scenario2	Scenario3	
Scenario1		0.028	0.178	0.206
Scenario2	0.038		0.032	0.07
Scenario3	0.136	0.034		0.170

E

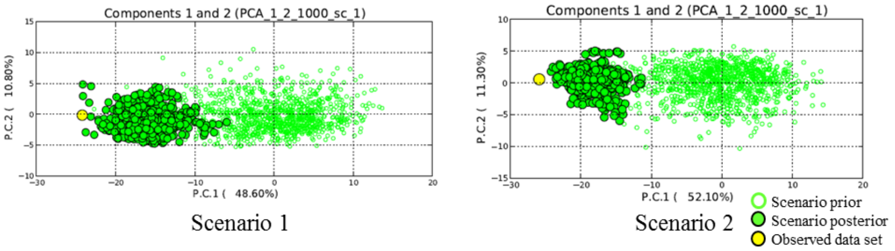

Supplementary Figure 3. ABC analysis to infer the best scenario by DIYABC. A: Graphic representation of three scenarios. A, B and C indicate the Mesoamerican genetic groups shown in Figure 2. Africa and Asia indicate African and Asian Jatropha. B: Prior and posterior distributions for scenario parameters. N1, N2, N2 and N4 indicate the effect sample size of African and Asian group, group A, B and C, respectively; t1 and t2 mean two historical divergence events. C: Logistic regression of posterior probabilities. D: Type I and Type II error rates for each scenario. E: Principal Component Analysis (PCA) of the model checking computation in scenario

1 and 2. Small green dots, large green dots, and yellow dots represent the prior distribution of parameters, the data set from the posterior predictive distribution, and the observed data, respectively.

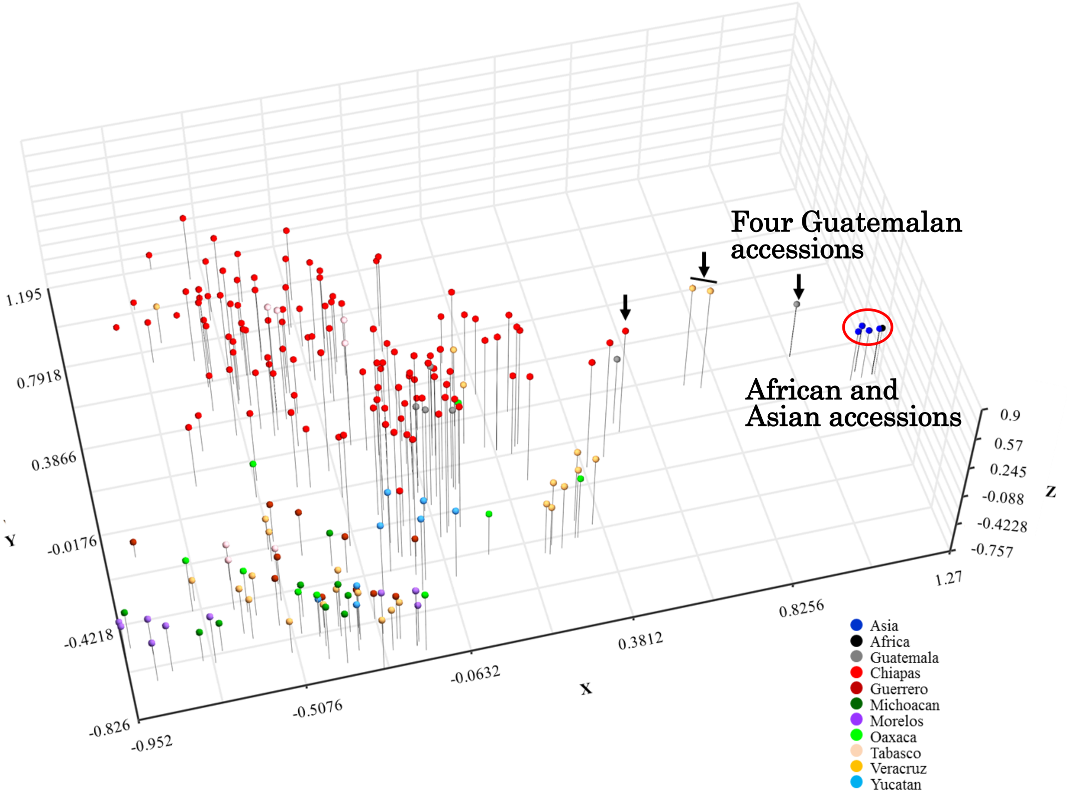

Supplementary Figure 4. Scatter plot for all accessions based on principal coordinate analysis (PCoA), in which 19.82% was explained by x axis, 10.12% was explained by y axis and 5.72% was explained by z axis. The black arrows indicate seven candidate accessions that share common ancestors of African and Asian accessions (see Supplementary Figure 2 and Figure 4). Four Guatemalan accessions are merged as one grey dot because of the same genotype. A red circle indicates African and Asian accessions.

1.2 Supplementary Tables

Supplementary Table 1. Genetic diversity of Jatropha from different geographical origins

Origin	Sample number	H_O	SE of H_O	H_E	SE of H_E	F_{IS}	Number of specific alleles
Africa	7	0.000	0.000	0.000	0.000	-	0
Cape Verde	1	0.000	0.000	0.000	0.000	-	0
Egypt	1	0.000	0.000	0.000	0.000	-	0
Madagascar	1	0.000	0.000	0.000	0.000	-	0
Sudan	2	0.000	0.000	0.000	0.000	-	0
Tanzania	1	0.000	0.000	0.000	0.000	-	0
Asia	32	0.003	0.002	0.002	0.001	-0.020	0
China	1	0.000	0.000	0.000	0.000	-	0
Indonesia	2	0.000	0.000	0.000	0.000	-	0
Philippines	7	0.000	0.000	0.000	0.000	-	0
Thailand	1	0.000	0.000	0.000	0.000	-	0
Vietnam	21	0.004	0.002	0.003	0.002	-0.031	0
Mesoamerica	207	0.158	0.011	0.306	0.019	0.487^{***}	161
Guatemala	9	0.081	0.019	0.177	0.025	0.582^{***}	0
Mexico	198	0.161	0.012	0.306	0.019	0.464^{***}	129
Chiapas	118	0.234	0.017	0.337	0.021	0.310^{***}	47
Central Chiapas	74	0.277	0.018	0.382	0.022	0.280^{***}	30
Centro†	27	0.278	0.018	0.363	0.021	0.253^{***}	10
Altos†	5	0.268	0.037	0.293	0.029	0.205^{***}	3
Fronteriza†	14	0.306	0.026	0.349	0.024	0.161^{***}	0
Frailesca†	15	0.235	0.021	0.366	0.023	0.388^{***}	2
Sierra†	13	0.298	0.024	0.328	0.025	0.131^{***}	1
Peripheral areas of Chiapas	44	0.161	0.022	0.212	0.022	0.251^{***}	1
Norte	2	0.037	0.016	0.140	0.027	0.857^{***}	0
Selva	3	0.129	0.021	0.237	0.024	0.600^{***}	0
Soconusco	34	0.173	0.027	0.178	0.026	0.043^{***}	0
Istmo-Costa	5	0.150	0.027	0.251	0.023	0.492^{***}	1
Guerrero	11	0.033	0.007	0.172	0.024	0.824^{***}	2
Michoacan	9	0.012	0.005	0.122	0.022	0.915^{***}	0
Morelos	10	0.016	0.007	0.123	0.025	0.879^{***}	0
Oaxaca	9	0.081	0.012	0.191	0.021	0.613^{***}	1
Tabasco	7	0.104	0.028	0.184	0.028	0.498^{***}	0

Veracruz	26	0.078	0.011	0.221	0.019	0.657***	23
Yucatan	8	0.008	0.004	0.064	0.020	0.894***	0

† Central Chiapas covering the Chiapas Central Depression, indicated by grey highlight. *** *P* < 0.001.

Supplementary Table 2. List of the SSR markers and primers used in the study.

SSR marker	Forward primer (5′-3′)	Reverse primer (5′-3′)
**JCG0001**	AGCACACCACCATTAAAGCC	GGCTTTGCATAACACCATCA
**JCG0007**	AATCGAAGAGCAGGTGCAGT	GCAAAATCAAGCCAAATCGT
**JCG0009**	TGGGCGATTGAGCTTACTTT	CCCTTCAATGGGTTCTTTCA
**JCG0027**	ATGATACGAGGACGAATGGG	GAATGTGGCTGCAGGGTATT
**JCG0028**	AGGTAACCAACAGTGACCCCT	CCGTCCCCTTTTATTGGATT
**JCG0034**	AGGTTGCACAGATACGGGAC	GCACAGTGACAAATTGTGCTT
**JCG0038**	GCATGCATTTTGGTTGTGAG	TGCAATGTAAGCCCATTCAA
**JCG0040**	TTGGGCTATAGTACCGCAGG	TTGAACTGAGGTGGAAACCC
**JCG0041**	TCCGTTGTTTGGTTGCAATA	GATGGTGGTTGCTCCAATCT
**JCG0050**	TCCTCGCCTTCAAGTGGTAA	GCTGCAGCAAGCACAATAAA
**JCG0054**	TCGACAAAACCCCAATTATCA	TCAGTTCACCCAACAGTCCA
**JCG0057**	AGCTTGCCTGAAAAACCAGA	TTTCAGCCACTACCTCAGCC
**JCG0061**	TCACTCTTCTGTCTCGCTTCC	CGCAAAATTTGTGTTGGAAT
**JCG0063**	CTTTGATGAGGCTTCCCTTG	CATCGCATCATCCAGTTGTC
**JCG0066**	TTTCCTGGTGTTTTGCATTG	TTCATCCCTTTCTGGTGGAG
**JCG0085**	CTCCCTTGTTCTTTGCTTCG	CAGCATATGACACCTGCACC
**JCG0087**	TGCTAAGGTCCCTCTTGGAA	TGAAGATGATGGGAAAAGGG
**JcGNS101**	CCTTTCTGTCATCTCCGAGG	CACGACACCATAAGCACTGG
**JcGNS105**	GACCCTCTGGCTGAAGTTTG	GCTGTCTGCAGCTCTGAATG
**JcGNS106**	CCTTCCCTCTTCCTCCAATC	CCTTCTCCTATACGCCATCG
**JcGNS0109**	TCGATGTCAGTACAGGCTGC	TTCTCCCTCTGTATGGTCGC
**JcGNS0112**	GGAAGACTGGAACTGCAAGC	TAGCCTACTGCAACAAGCCC
**JcGNS0113**	GGAGCGTAAGGATCTGCAAC	CTCTCTAAACCCTTGCAGGC
**JcGNS0117**	CTGCTGTCTCTGCTTGGTTG	GCACATTCACCCTCTCCTTC
**JcGNS0126**	GTGCCTGAAGTAAGGGCAAG	TCAGTGAGAGGCACAGTTGC
**JcGNS0127**	ATCCGTTCGACCTACACTGG	TTCACTAAGCCTCCGACCAC
**JcGNS0131**	GAAAGAGGCGAACAGTAGCG	GTTTCTTACGTCGCGTCCTC
**JcGNS0134**	TGGGTTTCAGCAGAATAGGG	TCTGCAGAGGCATCTGTGTC
**JcGNS0143**	ATGGTGCCATTGCTGATACC	AACTTGCTCCTAGGGCTTCC

**JcGNS0144**	AGAAGCCCATTGATGGTGTC	TCCTGCGTACTAATCCCAGC
**JcGNS0153**	CTTGGAAAGAGCTTCCATGC	ATGGGTGACTCTTCGTCTGC
**JcGNS0158**	CTTCGCTCGCTTTCTTGTTC	GATAGCTGCGGTGGAAAGAG
**JcGNS0165**	GTTGAAAACCCACAAGGACG	GAGCGAATGGCTAGGTTACG
**JcGNS0178**	TCAACAACCGCAACAACAAC	GGAAGAAAGCCAGCAGTGTC
**JcGNS0180**	ATTGGTTGTTGTTGTTGGGC	GGGAATAGAGAAAGCCGGAG
**JcGNS0182**	AAATGAGCTTGCAGCCTTTG	GTCGGGGGCCTTAAACTTAC
**JcGNS0183**	CCATTTTCGCTGTGTTGATG	CTAACCACTTGGGCCAGTTC
**JcGNS0187**	ACAACCCCAATGAAATGAGC	TATAGAGCTGGCATACCGCC
**JcGNS0189**	ACCCTTTTTGCCTTGTCATC	ACCTTCCAGGGAGACATTCC
**JcGNS0191**	TGCTCATCATCAAGCAATCC	GAAGTGGGTCTCCTTCAACG
**JcGNS0192**	TTATCCAAGCAACCAAACGG	TAGCCGCTTCTCTCTCTTCG
**JcGNS0193**	TCATGATGAGAATCTTGCGG	CCAAGCGAGACAAAGGAGAG
**JcGNS0196**	TCTTGAATGGAGAAATGGGC	CCCCTCTCTATTTTCCCCAG
**JcGNS0204**	CATGGCAACCAAGAACATTG	TGGGATAGGCAGAGTCCAAC
**JcGNS0214**	CTTCTTCTGTCCAAGGGAGC	TTTGAGATGCCCAAGAGAGG
**JcGNS0217**	TATACCTGCCTCTATGGGCG	AAAGGGTAAGGTTGAACGGG
**JcGNS0224**	CGATGGACGTAAAGCTCACC	TTGCCCTTAAACTGCCTCAC
**JcGNS0225**	GTGACAGGGTATGTGCATGG	ATTGTGTTGGAGGCTGAAGG
**JcGNS0228**	GCTCAAAGCTCACCATCTCC	ATGCTATCCATTCTGCTGGC
**JcGNS0233**	GGGAGCCTGAGGAAAGAATC	GGCAAAAACCCAACTTAGCC
**JcGNS0241**	GGACGTGGTTCAGTCATGTG	ACTTCCATCCTTCGCCTTTC
**JcGNS0245**	CCAACGAGGACCGATGTTAG	AAGTTAACCGGTGATGTCGC
**JcGNS0248**	CTGCTCCTGATTCCTTCTGG	TTTCCAAGTCCACTTGCTCC
**JcGNS0257**	GGATCTGCCTTTGATTCAGC	TCACCCCTCTTTCACCAATC
**JcGNS0261**	TGCACTTGGACTTGGTCTTG	CGTTTCACCAGTTTGACGAG
**JcGNS0270**	ATGTGCGGTCCTTTACCTTG	ATCTACAACCATTGGGCAGC
**JcGNS0279**	AGATTTGTGGGTTGGTCCAG	TGCGGTATAGTTGCATCCAG
**JcGNS0293**	CCGAGAAGTACAAACAGCAGAC	AACCCTCGTCGAACGATATG
**JcGNS0299**	GAGTTCCAACTGGGAAATGG	CCGAACAGGAATTCAACCAC

Supplementary Table 3. List of the RBIP markers and primers used in the study.

RBIP marker		LTR primer(5´-3´)		FLK primer(5´-3´)
**5S**	Left	GAGTGGGCACCGCACAACAA	Left	TGGAGAATTTGGGTTTGGTC
	Right	TTATTGCCGGGGCCTAACAC	Right	CTCGAGACCTCTCAACGAAC
**8A**	Left	GAAAATTAAATCCAACAATA	Left	GAACCAGGATCACGTTCAAC
	Right	TGAGATTAATTCTTACATAT	Right	TCGCCCCACTTACTTTCTTG
**8B**	Left	GAAAATTAAATCCAACATGT	Left	CAAAGCACACGAGGATTCAG
	Right	GAGATTAATTCTTAACAGAA	Right	CAGGTCCAAATCTCCTCGTG
**9A**	Left	TTTCTCTTCATCCGACAAAA	Left	AGATGCTGATAGGGTTGGTG
	Right	ATTTTTCCCTCTGTGAAACAG	Right	CAGCACGGCCTCGTTTATAG
**9B**	Left	TTTCTCTTCATCCGACATGG	Left	GTGGGATCTTGAAGGACCAG
	Right	TTTCCCTCTGTGAAACACCC	Right	TGTTGAGAAACATGGTCAAGC
**9C**	Left	TTTCTCTTCATCCGACAAAA	Left	TTGCCCAAATTTCACTTCATC
	Right	TTTCTCTCTGTAAAACATCT	Right	CCGAATTTTGAGCCAGCTTG
**9D**	Left	TTTCTCTTCATCCGACATTA	Left	CTTACTGACTTCATTAATTG
	Right	TTTCCCTCTGTGAAACAGAT	Right	CACCCACCCTCTTCTTCATC
**11SA**	Left	ACAACTAATCCTTAACAAAC	Left	CACTCCTAAAATGCGGCTAAC
	Right	CCCACATTAAATCTACAAGG	Right	TTCCACTGCTATTGTTTAATTCAT

Supplementary Table 4. Variation in agronomic traits among three regions and among three genetic groups.

Yield related traits	Geographic region						Genetic groups					
	Central Chiapas		Peripheral areas of Chiapas		Other Mexican states		A		B		C	
	Mean	SD	Mean	SD	Mean	SD	Mean	SD	Mean	SD	Mean	SD
Number of inflorescences per plant*	18.55	13.24	15.14	11.84	9.38	7.71	18.36	13.04	13.64	11.54	8.79	6.61
Number of female flowers per plant*	76.52	63.94	66.82	49.82	49.91	40.42	73.75	61.78	61.11	49.21	49.18	43.14
Ratio between female and male flowers	24.17	43.53	7.21	26.21	5.57	25.75	17.90	38.98	11.42	32.08	7.24	26.20
Seed yield (g) per plant*	70.57	65.07	55.50	62.07	51.56	47.49	72.12	64.31	55.05	58.92	43.05	44.52

* Traits correlated with each other ($P < 0.001$)

Supplementary Table 5. H_O, H_E, and F_{IS} of Groups A, B, and C

Group	H_O	H_E	F_{IS}
A	0.290	0.405	0.289***
B	0.120	0.210	0.432***
C	0.042	0.170	0.754***

*** $P < 0.001$.

Supplementary Table 6. The genetic distance (Nei's D_A distance) between Groups A, B, C, and African and Asian accessions

Group	Africa and Asia
A	0.255
B	0.120
C	0.220

Supplementary Table 7. The genetic distance (Nei's D_A distance) among collection sites

Origin		Africa	Asia	Guatemala	Chiapas		Guerrero	Michoacan	Morelos	Oaxaca	Tabasco	Veracruz
					Central	Peripheral						
Asia		0.00										
Guatemala		0.09	0.09									
Chiapas	Central	0.22	0.21	0.12								
	Peripheral	0.15	0.15	0.06	0.05							
Guerrero		0.22	0.21	0.13	0.12	0.09						
Michoacan		0.24	0.24	0.15	0.15	0.10	0.05					
Morelos		0.26	0.25	0.18	0.17	0.11	0.06	0.03				
Oaxaca		0.18	0.17	0.11	0.10	0.06	0.04	0.05	0.05			
Tabasco		0.28	0.27	0.18	0.15	0.13	0.16	0.14	0.15	0.13		
Veracruz		0.16	0.16	0.10	0.09	0.06	0.05	0.06	0.08	0.04	0.13	
Yucatan		0.20	0.20	0.13	0.15	0.09	0.07	0.06	0.09	0.07	0.16	0.07

Supplementary Table 8. Numbers of accessions from different collection sites, characterized by the number of retrotransposons (RTN)

Number of RTNs	Number of Accessions										
	Africa	Asia	Guatemala	Chiapas	Guerrero	Michoacan	Morelos	Oaxaca	Tabasco	Veracruz	Yucatan
**8**	7	34	1	2	0	0	0	0	0	2	0
**7**	0	0	6	5	0	0	0	2	0	7	0
**6**	0	0	0	32	2	0	0	2	0	5	0
**5**	0	0	0	36	5	2	5	4	2	8	5
**4**	0	0	0	21	2	5	0	1	3	4	0
**3**	0	0	0	8	0	0	3	0	0	0	0
**2**	0	0	0	3	0	0	0	0	0	0	0
**1**	0	0	0	0	0	0	0	0	0	0	0
**0**	0	0	0	0	0	0	0	0	0	0	0
**Total**	7	34	7	107	9	7	8	9	5	26	5
**Average**	8.00	8.00	7.14	5.02	5.00	4.29	4.25	5.56	4.4	5.81	5.00
